# Kinetic investigation of calcium-induced Sorcin aggregation by stopped-flow light scattering

**DOI:** 10.1042/BCJ20253194

**Published:** 2025-10-28

**Authors:** Qiushi Ye, Kathleen Joyce Carillo, Nicolas Delaeter, Lei Zhang, Jaekyun Jeon, Yanxin Liu

**Affiliations:** 1 School of Physics, Xi’an Jiaotong University, Xi’an, Shaanxi, 710049, China; 2 Institute for Bioscience and Biotechnology Research, University of Maryland | National Institute of Standard and Technology, Rockville, MD, 20850, U.S.A.; 3 Department of Chemistry and Biochemistry, University of Maryland, College Park, MD, 20740, U.S.A.

**Keywords:** calcium binding protein, multidrug resistance, protein aggregation, Sorcin, stopped-flow

## Abstract

Sorcin, a penta-EF hand calcium-binding protein, is implicated in multidrug resistance (MDR) in various cancers and has roles in neurodegenerative diseases. It regulates cellular calcium homeostasis by interacting with calcium channels, pumps, and exchangers in a calcium-dependent manner. Calcium binding induces a conformational change in Sorcin, exposing hydrophobic surfaces that mediate protein interactions and calcium flux between the cytosol and endoplasmic reticulum. These exposed surfaces can also drive Sorcin aggregation in the absence of binding partners. Here, we exploited calcium-induced conformational changes and aggregation of Sorcin as a model to study its calcium sensitivity and aggregation mechanisms. Stopped-flow light scattering revealed that Sorcin aggregation is reversible, co-operative, and primarily influenced by Sorcin concentration rather than physiological calcium levels. Our findings suggest that the calcium sensitivity of Sorcin is finely tuned by its expression level, highlighting its role as an intracellular calcium sensor. This work establishes Sorcin as a model system for studying protein aggregation mechanisms with implications for MDR and neurodegenerative diseases.

## Introduction

Sorcin was first identified as a **so**luble **r**esistance-related **c**alcium-binding prote**in** in the K562 leukemia cell line [1]. Subsequently, it was recognized as an overexpressed protein in several human tumors exhibiting multidrug resistance (MDR), including gastric, breast, and ovarian cancers, as well as glioblastoma [2–9]. The Sorcin gene resides in the same chromosomal region and amplicon as MDR-related genes, such as ATP-Binding Cassette Subfamily B Member 1 (MDR1)and ATP-Binding Cassette Subfamily B Member 4 (MDR3), highlighting Sorcin’s role in chemotherapy resistance [10]. Furthermore, a mitochondrial isoform of Sorcin interacts with the mitochondrial chaperone Trap1 (tumor necrosis factor receptor-associated protein 1) [11]. These proteins are co-upregulated in colorectal carcinomas and jointly protect cells against apoptosis induced by chemotherapeutic agents [11]. Beyond its role in cancer, Sorcin is overexpressed in brain tissue from Alzheimer’s and Parkinson’s patients, where it may counteract the elevated cytosolic calcium (Ca^2+^) concentrations associated with neurodegeneration by facilitating Ca^2+^ uptake into the endoplasmic reticulum [12]. Due to its roles in both cancer and neurodegeneration, Sorcin has emerged as a promising drug target for therapeutic intervention [13,14].

Beyond its involvement in human diseases, Sorcin plays a key role in regulating cellular Ca^2+^ homeostasis [15,16]. Upon Ca^2+^ binding, it undergoes a major conformational change that exposes hydrophobic surfaces, enhancing its interactions with other proteins [17–20]. In a Ca^2+^-dependent manner, Sorcin interacts with Ca^2+^ channels, pumps, and exchangers, including ryanodine receptors (RyRs), sarco/endoplasmic reticulum Ca^2+^ ATPase (SERCA), L-type voltage-dependent Ca^2+^ channels, and Na^+^-Ca^2+^ exchangers [21–25]. When cytosolic Ca^2+^ concentration is high, Sorcin inhibits Ca^2+^ release from the endoplasmic reticulum (ER) by interacting with RyRs while activating Ca^2+^ re-uptake through interactions with SERCA. This dual action promotes Ca^2+^ accumulation within the ER and reduces cytosolic Ca^2+^ levels, maintaining cellular Ca^2+^ balance [26].

**Figure 1:**
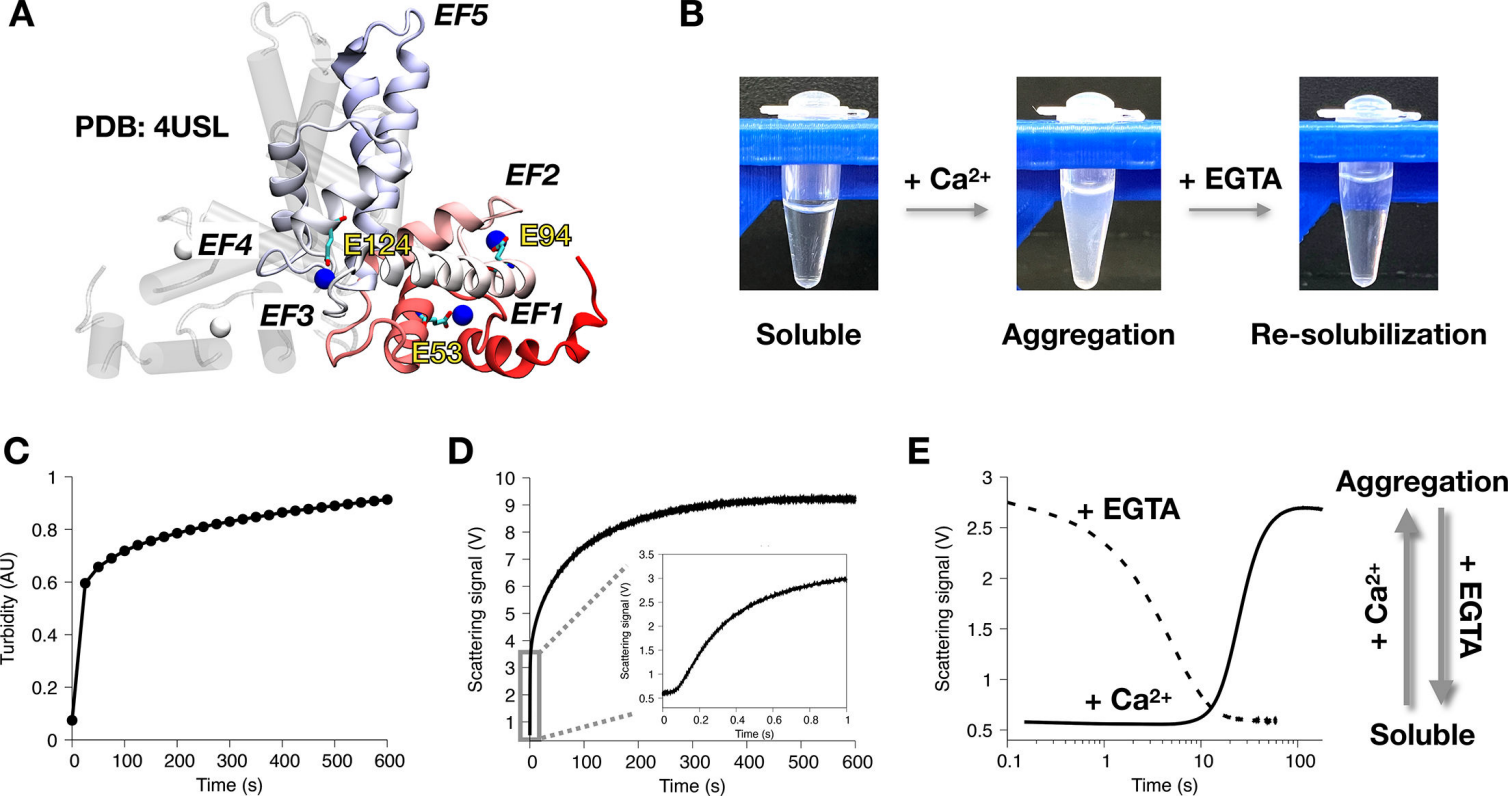
Calcium binding induces rapid and reversible Sorcin aggregation. (**A**) X-ray crystal structure of dimeric Sorcin in the Ca^2+^-bound state (Protein Data Bank: 4USL). Protomer A is shown as a transparent white cylindrical cartoon, and protomer B is shown as a ribbon cartoon colored from red (N-terminus) to blue (C-terminus). All five EF-hands and key Ca^2+^-binding residues are labeled. Bound Ca^2+^ ions in protomer B are shown as blue spheres. (**B**) Ca^2+^ binding triggers Sorcin aggregation, which is reversed by the addition of EGTA. (**C**) Sorcin aggregation is monitored using a turbidity assay that measures light scattering at λ=350 nm on a plate reader. (**D**) Stopped-flow light scattering experiments reveal fast initial aggregation kinetics with a ~0.1 s lag phase. Light-scattering signals were converted to voltage using a photomultiplier. The experiments were conducted by rapidly mixing 100 µM Ca^2+^ with 10 µM Sorcin, under the same conditions as the plate reader-based turbidity assay. (**E**) Reversal of Ca^2+^-induced Sorcin aggregation monitored by stopped flow light scattering. Aggregation of 1 µM Sorcin is induced by 150 µM Ca^2+^ with a 10 s lag phase (solid line) and reverses within 10 s upon adding 160 µM EGTA (dashed line). A schematic illustrating the reversible aggregation of Sorcin is shown on the right, highlighting its distinction from classical irreversible protein aggregation and amyloid fibril formation.

Sorcin is a 21.6 kDa penta-EF hand Ca^2+^-binding protein and functions as a homodimer (Figure 1A) [18]. Structurally, it consists of a glycine-rich N-terminal domain (NTD, residues 1–32) and a Ca^2+^-binding C-terminal domain (CBD, residues 33–198). X-ray crystallography and AlphaFold predictions show that the NTD is flexible in the absence of Ca^2+^ and may interact with the CBD upon Ca^2+^ binding [15,18]. Our recent structural characterizations of full-length Sorcin using NMR indicate that the NTD predominantly adopts an α-helical structure [27]. The CBD comprises five EF-hand motifs. Structural studies have also revealed that Ca^2+^ binding to EF-hands 1–3 induces a concerted reorientation of the central D-helix, exposing hydrophobic surfaces that mediate target recognition and membrane association [15]. The Ca^2+^-free crystal structure of the Sorcin Ca^2+^-binding domain established a stable EF4–EF5 dimerization interface, whereas subsequent Ca^2+^-bound structures demonstrated a ~20° rotation of the EF1–EF3 subdomain, identifying EF3 as the lowest affinity but principal regulatory site [15,16,18]. These conformational transitions provide a structural basis for Ca^2+^-dependent partner binding and suggest that, in the absence of physiological partners, the newly exposed hydrophobic surfaces may promote self-association.

Building on this framework, the present study uses stopped-flow light scattering to quantify the kinetics and co-operativity of Ca^2+^-induced Sorcin aggregation, providing a mechanistic link between structural activation and reversible self-assembly. We further exploit Ca^2+^-induced Sorcin conformational change and resulting aggregation to investigate its Ca^2+^ sensitivity and aggregation mechanisms. Protein aggregation has predominantly been studied using fluorescence spectroscopy with aggregate-sensitive dyes, such as thioflavin-T, or through turbidity assays that measure reduced transmitted light due to scattering. However, the limited temporal resolution of instruments like plate readers has made it challenging to capture rapid aggregation kinetics, particularly the early stages such as nucleation. The stopped-flow technique, widely used to monitor rapid chemical reactions, including protein folding on millisecond timescales, has significant potential for light scattering applications, though it had remained underexplored [28]. Recently, Jeon et al. demonstrated the use of stopped-flow light scattering to study the aggregation of amyloid-β (Aβ) peptides, providing quantitative analysis of Aβ oligomeric states over extended timescales, ranging from milliseconds to hours [29].

Using stopped-flow light scattering, we observed that Sorcin aggregation is reversible and co-operative, with minimal sensitivity to physiological Ca^2+^ levels but high sensitivity to Sorcin concentration. Our results indicate that Sorcin’s Ca^2+^ sensitivity is finely tuned by its expression level, providing new insights into its role as an intracellular Ca^2+^ sensor and its contribution to MDR. These findings establish Sorcin as a robust model system for investigating protein aggregation mechanisms, with implications for both cancer resistance and neurodegenerative disease research.

## Results

### Ca^2+^-induced Sorcin aggregation is reversible

We confirmed that Ca^2+^ binding induces Sorcin aggregation, as previously reported [17], by mixing Ca^2+^ with Sorcin in a test tube. Upon addition of Ca^2+^, the initially clear Sorcin solution visibly turned turbid, indicating aggregation (Figure 1B). When EGTA, a Ca^2+^ chelator, was subsequently added, the turbid Sorcin/Ca^2+^ mixture reverted to a clear solution, demonstrating that Sorcin aggregation is fully reversible upon Ca^2+^ removal through chelation by EGTA (Figure 1B). To monitor the aggregation process quantitatively, we utilized a turbidity assay, measuring light scattering at 350 nm. Aggregation kinetics were assessed using a plate reader (Figure 1C). While this approach captured the final equilibrium state, the initial aggregation phase occurred too rapidly to be resolved due to the plate reader’s limited time resolution (Figure 1C).

To resolve these rapid kinetics, we employed stopped-flow light scattering, ideal for capturing reactions on the millisecond (ms) to second (s) timescale [29]. In our setup, a 565 nm light-emitting diode (LED) illuminated the sample, and scattered light was detected by a photomultiplier tube (PMT) at a 90° angle. Aggregation was recorded as a change in voltage (V) detected by the PMT. Under the same conditions as the turbidity assay (10 µM Sorcin with 100 µM Ca^2+^), aggregation kinetics were recorded with a time resolution of 60 ms, as shown in Figure 1D. Consistent with turbidity assay results, aggregation reached a plateau within 10 minutes. Additionally, a lag phase of ~0.1 s preceded the rapid growth phase, confirming that our stopped-flow instrumental setup is well suited to study fast Sorcin aggregation kinetics.

With the stopped-flow light scattering implemented, we further investigated the re-solubilization rate of aggregated Sorcin upon EGTA addition. The highly sensitive PMT revealed that 1 µM Sorcin with 150 µM Ca^2+^ reached a plateau of 2.75 V with a 10-s lag (Figure 1E). Pre-incubated 1 µM Sorcin and 150 µM Ca^2+^ yielded the same signal of 2.75 V, but the addition of 160 µM EGTA rapidly dissolved the aggregates within 10 s (Figure 1E), confirming the reversibility of Ca^2+^-induced Sorcin aggregation and showing that re-solubilization occurs as quickly as aggregation.

### Effect of protein concentration on Sorcin aggregation

Using stopped-flow light scattering, we observed that Sorcin aggregated at a concentration of 1 µM (Figure 1E), ten times lower than in our plate reader-based turbidity assays (Figure 1C). However, to induce aggregation at this low Sorcin concentration, a higher Ca^2+^ concentration (150 µM) was needed, resulting in a lag time approximately 100-fold longer than with 10 µM Sorcin. These results suggest that protein concentration influences Sorcin aggregation kinetics, a dependence commonly observed in protein aggregation.

**Figure 2:**
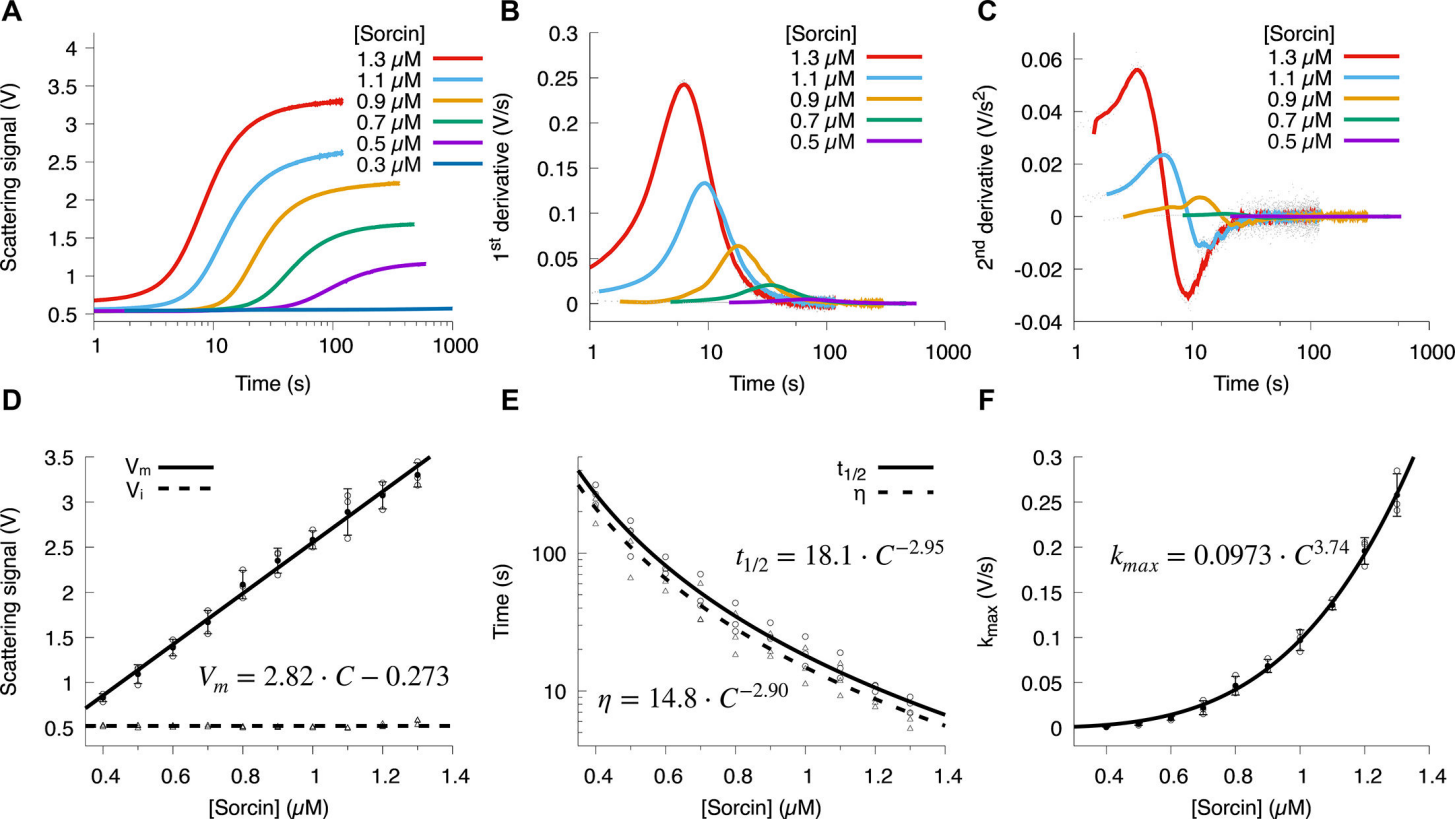
Sorcin aggregation kinetics depend on protein concentration. (**A**) Representative aggregation kinetics curves at varying Sorcin concentrations. (**B**) The first derivative of these curves is presented. (**C**) The second derivative, with raw data shown as gray dots and a running average (solid line) to reduce noise. (**D**) The signal plateau (V_m_), representing the maximum extent of aggregation, scales linearly with protein concentration, while initial signal (V_i_) remains constant. Extracted V_m_ and V_i_ values are shown as circles and triangles, respectively, with linear and horizontal fits depicted as solid and dashed lines. (**E**) The aggregation half-time (t_1/2_) and the time at which the aggregation reaches its maximum rate (*η*) both follow a power law with a scaling exponent of ~3. t_1/2_ and *η* values from individual experiments are shown as circles and triangles, and power-law fits as solid and dashed lines, respectively. (**F**) The maximum aggregation growth rates (k_max_) depend on Sorcin concentration and were fitted to a power-law model (solid line). Data were collected from three independent replicates at each Sorcin concentration. The extracted kinetic parameters were plotted and fitted in panels (D–F) to illustrate the experimental variability.

To further explore protein concentration effects, we varied Sorcin concentrations from 0.3 µM to 1.3 µM in stopped-flow light scattering with a fixed 150 µM Ca^2+^ concentration. Representative aggregation traces are shown in Figure 2A. The first and second derivatives of the aggregation kinetics curves (Figure 2B and C) reveal the maximum aggregation rate (k_max_) and the time (*η*) at which it occurs, respectively. As expected, higher Sorcin concentrations led to increased aggregation, indicated by higher final scattering plateaus and accelerated aggregation (Figure 2A). Fitting the data with a double-exponential function (see Methods and Supplementary Figure S1), we found that aggregate levels, quantified by the maximum scattering signal (V_m_), increased linearly with Sorcin concentration, with a slope of 2.82 ± 0.08 V/µM (Figure 2D).

The double-exponential fit also yielded other kinetic parameters, including the aggregation half-time (t_1/2_) and two rate constants. As Sorcin concentration increased, t_1/2_ shortened according to a power law, $t_{1\text{/}2}=18.1\pm 2.49\times C^{\left(-2.95\pm 0.17\right)}$ , indicating a complex aggregation mechanism potentially involving secondary nucleation or growth (Figure 2E). Both k_fast_ and k_slow_ also increased with Sorcin concentration (Supplementary Figure S2A).

We calculated the k_max_ and the time (*η*) at which it occurs by taking derivatives of the kinetics trace (Figure 2B and C). The dependence of *η* on Sorcin concentration followed a similar power law $\text{η = 14.9 ± 2.59×}{\text{C}}^{\left(\text{-2.90 ± 0.21}\right)}$ , aligning with the t_1/2_ exponent, validating our double-exponential fit approach (Figure 2E). The observed *η* was consistently shorter than t_1/2_ at each protein concentration, suggesting a fast initial process followed by slower growth, reinforcing the need for a double-exponential fit (Figure 2E). k_max_ also followed a power law ${\text{k}}_{\text{max}}\text{ = 0.0973 ± 0.002×}{\text{C}}^{\left(\text{3.74 ± 0.08}\right)}$ as protein concentration increased (Figure 2F), and higher Sorcin concentrations decreased lag time 𝜏 (Supplementary Figure S2B).

### Effect of Ca^2+^ concentration on Sorcin aggregation

**Figure 3:**
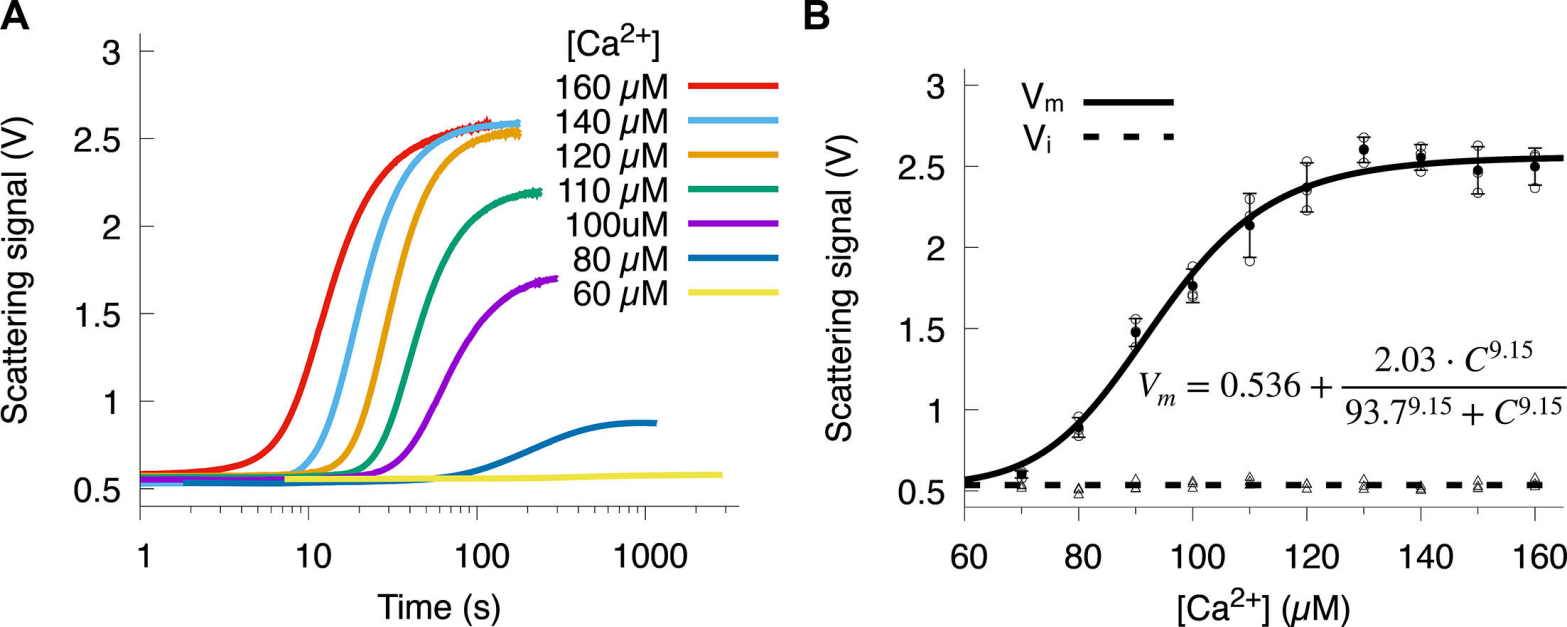
Ca^2+^ concentration dependence of the Sorcin aggregation kinetics. (**A**) Representative aggregation kinetics curves at varying Ca^2+^ concentrations. (**B**) The dependence of V_m_ on Ca^2+^ concentration follows a sigmoidal response. V_m_ and V_i_ values from individual experiments are shown as circles and triangles, respectively. The relationship between V_m_ and Ca^2+^ concentration is fitted with a hill equation (solid line), while V_i_, which remains constant across different Ca^2+^ concentrations, is fitted with a horizontal line (dashed line). Data were collected from three independent replicates at each Ca^2+^ concentration. The extracted kinetic parameters were plotted and fitted in panels (**B**) to illustrate the experimental variability.

Given that Sorcin functions as a Ca^2+^ sensor, we expected that aggregation would depend on Ca^2+^ concentration. Indeed, higher Ca^2+^ concentrations led to more extensive and faster aggregation, as shown in Figure 3A. Unlike the linear dependence of V_m_ on Sorcin concentration, the V_m_ response to Ca^2+^ concentration was sigmoidal, best fit by a Hill equation, ${\text{V}}_{\text{m}}\text{ = }{\text{C}}^{\text{n}}\text{/\textbackslash{}(}{{\text{K}}_{\text{1/2}}}^{\text{n}}\text{ +}{\text{ C}}^{\text{n}}\text{\textbackslash{})}$ as shown in Figure 3B. A Hill coefficient of *n*=9.15 ± 0.89 indicates high co-operativity in Ca^2+^-induced aggregation, with half of Sorcin aggregating at K_1/2_=93.7 ± 1.10 µM Ca^2+^. Higher Ca^2+^ concentrations also shortened all aggregation timescales, including t_1/2_, *η*, and *τ*, and accelerated all kinetic rates (Supplementary Figure S3).

The co-operative activation process observed is consistent with interdependent EF-hand dynamics. The sigmoidal response and elevated Hill coefficient indicate that Ca^2+^ binding at one site enhances the affinity of neighboring sites, likely through the structural coupling of EF1–EF3 across the central D-helix. Structural studies have shown that EF1–2 exhibits the high Ca^2+^ affinity, and EF3 Ca^2+^ binding drives a global rotation of the D-helix [15]. This cascade provides a plausible molecular basis for the co-operative kinetics observed here: once EF1–2 are occupied, the subsequent binding of Ca^2+^ to EF3 amplifies hydrophobic surface exposure, triggering rapid self-association. The plateau in aggregation rate at higher Ca^2+^ levels suggests that once the EF1–EF3 module is fully activated, additional Ca^2+^ no longer increases the extent of conformational opening or aggregation propensity. These findings highlight that Sorcin’s aggregation reflects its intrinsic allosteric regulation by Ca^2+^, a mechanism that balances sensitivity to transient Ca^2+^ signals with structural reversibility under physiological conditions.

### Mg^2+^ inhibition of Ca^2+^-induced Sorcin aggregation

**Figure 4:**
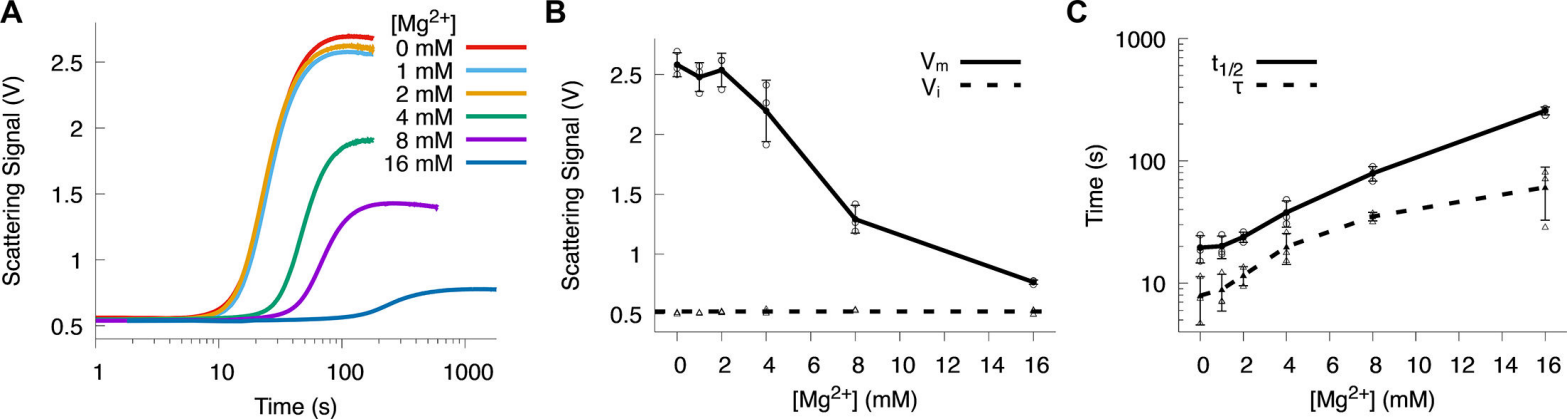
Effect of Mg^2+^ on Sorcin aggregation. (**A**) Representative aggregation kinetics curves at varying Mg^2+^ concentrations. (**B**) The dependence of V_m_ on Mg^2+^ concentration. (**C**) The dependence of the aggregation half-time (t_1/2_) and lag time (*τ*) on Mg^2+^ concentration. Data were collected from three independent replicates at each Mg^2+^ concentration. The extracted kinetic parameters were plotted from each experiment in (**B**) and (**C**). The averages were calculated from these three replicates and connected by lines to show the trend. The standard deviations were shown as the error bar.

Sorcin can also bind Mg^2+^, which is present at higher cellular concentrations than Ca^2+^ in resting states. We examined how physiological Mg^2+^ levels affect Ca^2+^-induced Sorcin aggregation by repeating stopped-flow light scattering experiments (1 µM Sorcin with 150 µM Ca^2+^) in the presence of Mg^2+^. At <2 mM Mg^2+^, there was no effect on aggregation, but concentrations >4 mM significantly inhibited Ca^2+^-induced aggregation (Figure 4A–4C). The aggregation rates were shown in Supplementary Figure S4.

The fact that Mg^2+^ exerts an inhibitory influence on Ca^2+^-induced Sorcin aggregation suggests its competitive binding to EF-hand motifs and its role in stabilizing the protein’s inactive conformation. The hypothesis is well supported by the X-ray crystal structure of Sorcin bound with Mg^2+^, which resembles the apo state of Sorcin rather than the Ca^2+^-bound state (Protein Data Bank: 4U8D). Although Mg^2+^ can occupy EF-hand loops, its lower charge density prevents the large-scale conformational rearrangements characteristic of Ca^2+^ binding. As a result, Mg^2+^ maintains Sorcin in a compact, ‘closed’ state that limits exposure of hydrophobic surfaces necessary for intermolecular interactions. The partial suppression of aggregation at physiological Mg^2+^ concentrations therefore mirrors the in vivo environment, where Mg^2+^ serves as a stabilizing counterbalance to transient Ca^2+^ spikes. This interplay between Mg^2+^ and Ca^2+^ underscores Sorcin’s finely tuned allosteric control: while Ca^2+^ binding drives activation and self-association, Mg^2+^ binding biases the equilibrium toward the inactive state, thereby preventing constitutive aggregation under resting conditions.

### Effect of salt concentration on Sorcin aggregation

**Figure 5:**
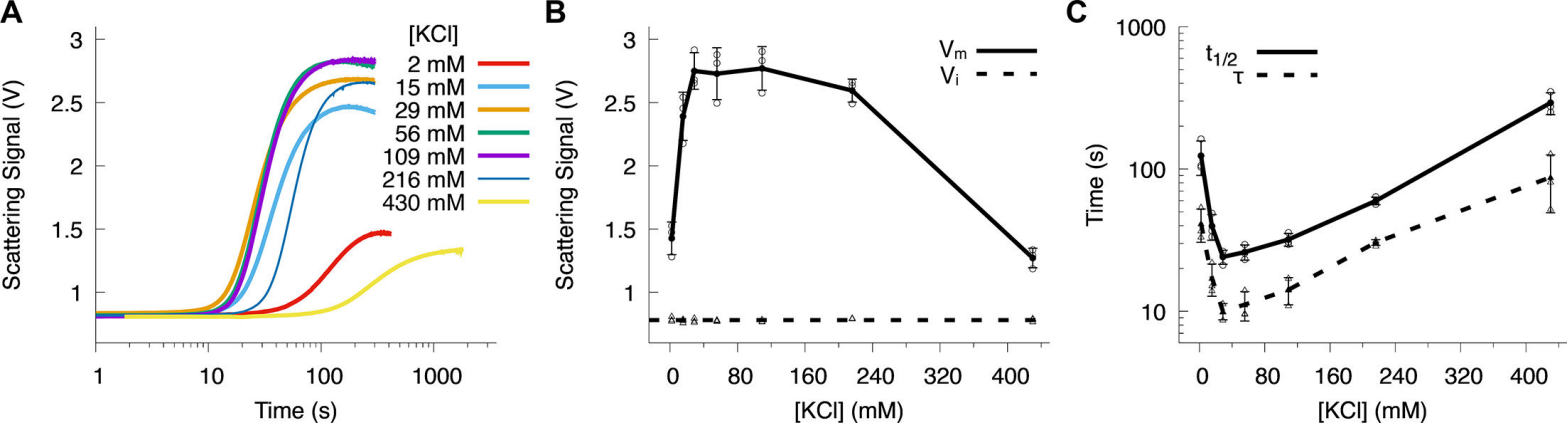
Effect of salt concentrations on Sorcin aggregation. (**A**) Representative aggregation kinetics curves at varying KCl concentrations. (**B**) The dependence of V_m_ on KCl concentration. (**C**) The dependence of the aggregation half-time (t_1/2_) and lag time (*τ*) on KCl concentration. Data were collected from three independent replicates at each KCl concentration. The extracted kinetic parameters were plotted from each experiment in (**B**) and (**C**). The averages were calculated from these three replicates and connected lines to show the trend. The standard deviations were shown as the error bar.

Salt concentration can affect protein aggregation by screening charges and weakening protein–protein interactions. To test this effect, we varied KCl concentration from 2 mM to 430 mM. High salt levels inhibited aggregation, as reflected by lower V_m_ (Figure 5A and B), increased t_1/2_ (Figure 5C), and slower rates (Supplementary Figure S4). Surprisingly, very low salt concentrations (<30 mM) also inhibited aggregation, potentially by stabilizing intramolecular interactions in the Ca^2+^-free state of Sorcin. This stabilization could increase the energy barrier for transitioning to the Ca^2+^ bound, aggregation-prone state. Further studies are needed to elucidate the molecular mechanisms behind this unusual salt dependence.

We hypothesize that changes in ionic strength modulate Sorcin aggregation by altering the balance of electrostatic and hydrophobic interactions that stabilize the Ca^2+^-bound state. Increasing salt concentration weakens long-range electrostatic attractions while enhancing the relative contribution of short-range hydrophobic forces, resulting in reduced aggregation amplitude and slower kinetics. This sensitivity suggests that ionic shielding can dampen the association between exposed hydrophobic patches on neighboring monomers, effectively raising the energetic threshold for nucleation. In physiological contexts, the cytosolic ionic milieu thus acts as an additional regulatory layer, constraining Sorcin’s self-association to specific microenvironments, such as near membranes or Ca^2+^ release sites, where local ionic strength or dielectric conditions may transiently favor intermolecular contacts.

### Effect of EF-hand mutations on Sorcin aggregation

Sorcin is a penta-EF-hand protein, but only the first three EF hands bind Ca^2+^ due to sequence mutations in EF4 and EF5 [30]. We tested point mutations in EF1–3 (E53Q, E94A, and E124A) to assess their impact on Ca^2+^-induced aggregation. Each mutation was sufficient to abolish aggregation at basal conditions (1 µM Sorcin and 150 µM Ca^2+^) (Supplementary Figure S5), confirming their effectiveness in disrupting Ca^2+^ binding.

**Figure 6:**
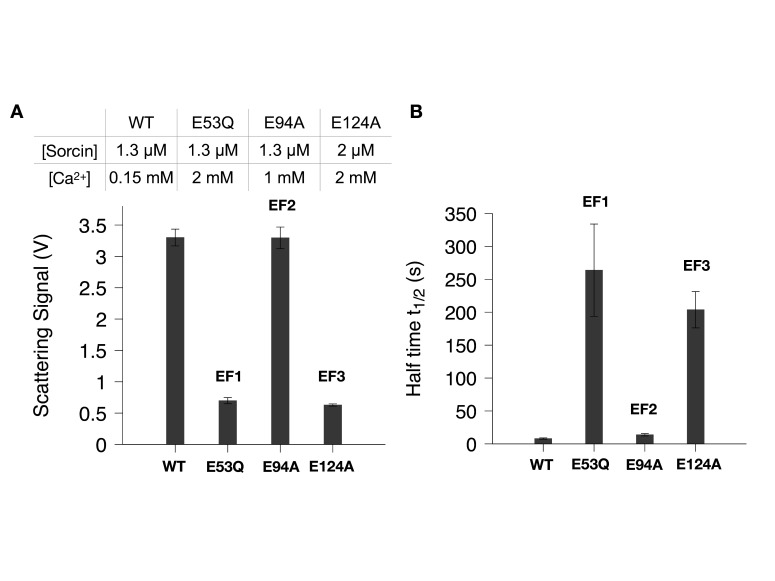
Effect of mutations in three EF hands on Sorcin aggregation. (**A**) Effect of the mutations on V_max_. (**B**) Effect of the mutations on the aggregation half-time (t_1/2_). Note that all aggregation assays for the mutations were performed at either higher Sorcin or Ca^2+^ concentrations compared with the wildtype experiments, as indicated in the table. No aggregation was observed for the mutations under the conditions used for the wildtype aggregation experiments (see Supplementary Figure S5). The averages and the standard deviations were calculated from three independent replicates.

Increasing protein or Ca^2+^ concentrations partially restored aggregation for specific mutants, as shown in Figure 6. With 1.3 µM Sorcin and 150 µM Ca^2+^, wildtype Sorcin aggregated rapidly (V_m_=3.3 V, t_1/2_=8 s), while none of the mutants aggregated. At 1 mM Ca^2+^, the EF2 mutant (E94A) reached a V_m_ similar to wild-type with slower aggregation kinetics (t_1/2_=14 s). Even with 2 mM Ca^2+^, the EF1 mutant (E53Q) still aggregated less and even slower (V_m_=0.7 V, t_1/2_=264 s) than wild-type and the EF2 mutant (E94A) in the presence of 1 mM Ca^2+^. The EF3 mutant (E124A) required 2 µM protein and 2 mM Ca^2+^ to show mild aggregation (V_m_=0.6 V, t_1/2_=204 s). These findings suggest co-operative interactions among EF hands, with each EF hand contributing differentially to Ca^2+^ sensitivity and aggregation.

## Discussions

A major function of Sorcin is to regulate intracellular Ca^2+^ levels by interacting with Ca^2+^ channels and pumps on the ER membrane [21–25]. This regulation is initiated by Sorcin’s ability to sense changes in Ca^2+^ concentration: when Ca^2+^ levels rise, Sorcin binds Ca^2+^ via its EF-hand motifs, triggering a conformational change that exposes hydrophobic residues on its surface [15]. This exposed hydrophobic region mediates Sorcin’s interactions with Ca^2+^ channels and pumps, ultimately suppressing Ca^2+^ release from channels and activating pumps to sequester excess cytosolic Ca^2+^ back into the ER [31]. In the absence of binding partners, Ca^2+^-bound Sorcin self-associates through these hydrophobic surfaces, leading to protein aggregation in vitro [18]. The aggregation kinetics observed here align closely with the conformational mechanisms elucidated by structural studies of Sorcin [15,16,18]. The rapid, Ca^2+^-dependent increase in light scattering likely reflects the exposure of hydrophobic patches upon EF-hand activation, particularly the EF3-driven rotation of the D-helix that repositions the EF1–EF3 module relative to the rigid EF4–EF5 dimeric scaffold. In this view, aggregation arises from the same Ca^2+^-induced exposed surfaces that normally engage protein partners such as the RyR [18].

A unique feature distinguishing Sorcin from other aggregation-prone proteins like Aβ peptides and α-synuclein is its reversible aggregation [17,32,33]. Ca^2+^-induced Sorcin aggregation was fully reversible upon Ca^2+^ removal with EGTA, as demonstrated in Figure 1. Remarkably, the disaggregation rate was as fast as the aggregation process, suggesting that Sorcin aggregates form via interactions between well-folded proteins rather than by unfolded or misfolded proteins. This characteristic contrasts with most well-studied aggregation systems, where aggregation is often linked to misfolding or alternative structural states. This reversibility of aggregation likely originates from the reversible nature of Ca^2+^-induced conformational changes of Sorcin, supporting a model in which Sorcin dynamically interconverts between dimeric, oligomeric, and aggregated states rather than forming irreversibly misfolded assemblies. Integrating these structural insights thus situates our kinetic observations within a unified model of Ca^2+^-regulated Sorcin activation, in which conformational exposure of interacting surfaces drives both functional partner binding and reversible self-association in the absence of binding partners. Sorcin thus provides a unique system for studying reversible aggregation kinetics and thermodynamics, offering insight relevant to understanding aggregation in pathological contexts.

As a cellular Ca^2+^ sensor, a key question about Sorcin is its sensitivity to Ca^2+^ concentrations. Although EF-hands 1 and 2 bind Ca^2+^ with μM affinity, no Sorcin aggregation was observed at Ca^2+^ concentrations in the μM range. This suggests that either Sorcin conformational change is not triggered by Ca^2+^ binding to EF-hands 1 and 2, or that Sorcin responds to μM Ca^2+^ concentrations through a mechanism other than hydrophobic surface exposure. Aggregation was only observed at Ca^2+^ concentrations above 60 μM, with half-maximal aggregation at 93 μM, suggesting that EF-hand 3 may have a Ca^2+^ affinity in the tens of μM range (Figure 3). The result renders Sorcin as a much less sensitive Ca^2+^ sensor than the well-studied Ca^2+^-binding protein calmodulin, which has sub-micromolar and micromolar Ca^2+^ affinity [34]. Moreover, the presence of mM levels of Mg^2+^, typical in cellular environments, may increase Ca^2+^ binding affinity to the hundreds of μM range (Figure 4A–4C). This relatively high Ca^2+^ concentration required to trigger Sorcin aggregation is indeed puzzling and counterintuitive. However, there are physiological contexts in which such elevated local Ca^2+^ levels may occur. Although resting cytosolic Ca^2+^ concentration is typically below 1 µM, transient microdomains near Ca^2+^ channels (e.g. RyRs and voltage-gated Ca^2+^ channels) can reach tens to hundreds of micromolar during excitation or stress responses. These localized Ca^2+^ spikes could transiently promote Sorcin aggregation and disaggregation cycles, particularly in excitable cells such as cardiomyocytes and neurons, where Sorcin is highly expressed.

While Sorcin aggregation shows limited sensitivity to Ca^2+^, it is highly responsive to Sorcin protein concentration. Increasing Sorcin levels from 0.4 μM to 1.3 μM decreased the aggregation half-time by an order of magnitude, from ~300 s to ~10 s (Figure 2). This high sensitivity to Sorcin concentration may allow cells to fine-tune Ca^2+^ responses by modulating Sorcin expression. The concentration dependence of Sorcin aggregation observed here likely reflects physiological mechanisms regulating the association to itself and binding partners in cells. Although precise intracellular concentrations are not well defined, proteomic analyses suggest that Sorcin is highly abundant in several tissues and cell types, including cardiomyocytes, neurons, and multidrug-resistant cancer cells, as found in PaxDb [35]. Estimated cytosolic levels in the submicromolar to micromolar range overlap with the concentrations examined in our experiments (0.3–1.3 µM), supporting the physiological relevance of our findings. Given Sorcin’s high expression and its localization near Ca^2+^ release sites such as RyRs, local enrichment and transient increases in effective concentration could further promote reversible association in vivo. These results suggest that Sorcin’s concentration-dependent aggregation may represent a tunable, Ca^2+^-regulated mechanism for modulating its oligomeric state and functional interactions within excitable cells.

Additionally, Ca^2+^ sensitivity of Sorcin can be influenced by Mg^2+^ concentration (Figure 4) and ionic strength (Figure 5). Taken together, the combined effects of Ca^2+^ concentration, Mg^2+^ inhibition, and ionic strength illustrate how Sorcin’s self-association is governed by a delicate interplay between metal-ion binding and electrostatic regulation. Ca^2+^ binding initiates a co-operative cascade among EF1–EF3 that exposes hydrophobic interaction surfaces, while Mg^2+^ occupancy and high ionic strength stabilize the compact, inactive conformation by opposing those same structural transitions. This balance ensures that Sorcin remains soluble under basal cytosolic conditions yet can rapidly bind to target proteins or assemble into higher-order states in response to transient Ca^2+^ elevations or localized changes in the ionic environment. Such reversible regulation allows Sorcin to act as a dynamic Ca^2+^ sensor, switching between inactive, active, and self-associated forms, to fine-tune its roles in excitation–contraction coupling, vesicular trafficking, and stress response signaling.

Beyond elucidating Sorcin’s role in Ca^2+^ sensing and regulation, our study highlights Sorcin as a model system for understanding protein aggregation mechanisms. Unlike classical aggregation systems such as Aβ, α-synuclein, tau, Huntington protein with polyglutamine extensions, and prion protein, where aggregation studies typically require extended incubation under shaking or elevated temperatures, Sorcin aggregates rapidly and reversibly under easily controlled conditions. These features make Sorcin an ideal system for studying aggregation kinetics. Furthermore, Sorcin’s aggregation is highly tunable by protein, Ca^2+^, Mg^2+^, and ionic strength. The complex and co-operative nature of Sorcin aggregation revealed by stopped-flow light scattering provides a framework for investigating the fundamentals of protein aggregation, a process linked to various neurodegenerative diseases. Recent findings of Sorcin as an early biomarker in neurodegenerative conditions underscore the need for further mechanistic insights into its aggregation behavior [12].

## Conclusion

We implemented stopped-flow light scattering to investigate Sorcin’s rapid Ca^2+^-induced aggregation kinetics. Our results reveal that Sorcin aggregation is reversible, co-operative, and primarily driven by protein concentration rather than physiological Ca^2+^ levels. These findings highlight the critical role of Sorcin expression in modulating its Ca^2+^ sensitivity and aggregation behavior. This study advances our understanding of Sorcin’s function in Ca^2+^ homeostasis and establishes it as a valuable model system for exploring protein aggregation mechanisms, with potential relevance to MDR and neurodegenerative diseases.

## Methods

### Expression and purification of full-length human Sorcin

The DNA sequence encoding human Sorcin was codon-optimized for expression in *E. coli* and cloned into the pET151/D-TOPO vector, incorporating an N-terminal 6xHis tag followed by a Tobacco Etch Virus (TEV) protease cleavage site. *E. coli* Rosetta (DE3) cells were transformed with this construct and grown in Lysogeny broth with selective antibiotics. Cultures were incubated at 37°C until reaching an OD₆₀₀ of 0.5–0.6, at which point they were cooled to 16°C and induced with 0.3 mM isopropyl-β-D-thiogalactoside for protein expression overnight. Cells were harvested by centrifugation, resuspended in lysis buffer with 40 mM Tris-HCl pH 7.5, 480 mM KCl, 1 mM EGTA, 2 mM β-mercaptoethanol (βME), and 20 mM imidazole, and then lysed by sonication. The lysate was clarified by centrifugation, and the supernatant was purified using Ni^2+^-NTA affinity chromatography. The elution buffer contained 40 mM Tris-HCl pH 7.5, 150 mM KCl, 0.2 mM EGTA, 1 mM βME, and 300 mM imidazole. During overnight dialysis, the 6× His tag was cleaved by TEV protease, and imidazole was removed. The dialysis buffer contained 20 mM Tris-HCl pH 7.5, 80 mM KCl, and 1 mM DTT. Further purification was performed using anion-exchange chromatography (Mono Q column) followed by size-exclusion chromatography (Superdex 75 column). For anion-exchange chromatography, the buffer contained 20 mM Tris-HCl pH 7.5 and 1 mM DTT, with a KCl gradient increasing from 0 to 350 mM over 10 column volumes. Wildtype Sorcin eluted with approximately 130 mM KCl. The buffer used for size-exclusion chromatography contained 40 mM HEPES-KOH pH 7.5, 150 mM KCl, and 0.5 mM TCEP. Sorcin eluted at ~63 ml, consistent with a dimeric species. Protein purity was confirmed by SDS-PAGE, and concentration was determined by absorbance at 280 nm (*ε*=25,900 M^−^¹ cm^−^¹).

### Turbidity assay

The turbidity assay was conducted at 25°C in a 384-well microplate using a SpectraMax iD5 plate reader to monitor protein aggregation via light scattering at 350 nm. The final reaction mixture consisted of 10 µM Sorcin, 100 µM CaCl_2_, 40 mM HEPES-KOH pH 7.5, 150 mM KCl, and 1 mM βME. To initiate the aggregation reaction, CaCl_2_ was added as the final component.

### Stopped-flow light scattering

Stopped-flow light scattering experiments were carried out on an Applied Photophysics SX20 spectrometer, with data collected using Applied Photophysics Pro-Data SX software. Solutions of Sorcin and CaCl_2_, prepared in matched buffer, were pre-loaded into two syringes. For each measurement, 60 µl from each syringe was pneumatically driven into the 120 µl optical chamber and rapidly mixed. A 565 nm LED served as the light source, and light scattering was detected at a 90° angle by a PMT, with recorded voltage providing a measure of protein aggregation. Experiments were conducted at 22°C, with standard concentrations of 1 µM Sorcin and 150 µM CaCl_2_, unless otherwise specified in experiments. The buffer consisted of 40 mM HEPES (pH 7.5, adjusted with KOH), 150 mM KCl, and 1 mM βME, unless otherwise noted for KCl titrations. Titration experiments were conducted for Sorcin (0.3 µM to 1.3 µM), Ca^2+^ (60 µM to 160 µM), Mg^2+^ (0 to 16 mM), and KCl (0 to 450 mM). All measurements were performed in triplicate.

### Kinetic analysis

The time series data obtained from stopped-flow light scattering were analyzed by fitting a double-exponential function to extract the kinetic parameters of Sorcin aggregation:


(1) $$V\left(t\right)\;=\;V_{i}\;+\;(V_{m}-V_{i})\;/\;(1+0.5\;\times \exp^{k_{fast}\;\times \;\left(t_{1/2}-t\right)}+\;0.5\times \exp^{k_{slow}\;\times \;\left({\text{t}}_{1/2}-t\right)})$$

where V_i_ represents the initial background light scattering signal before aggregation begins, and V_m_ denotes the maximum light scattering signal, reflecting the final extent of aggregation. The parameter t_1/2_ is the aggregation half-time, at which the signal reaches the midpoint between V_i_ and V_m_. The rate constants k_fast_ and k_slow_ correspond to the fast and slow kinetic processes, respectively. The slope at t_1/2_ is defined as k_1/2_. The aggregation lag time (𝜏) was defined as the time at which V_i_ intersects with the tangent line, with slope k_1/2_, passing through the point [t_1/2_, (V_i_ + V_m_)/2] on the kinetic curve. Mathematically, 𝜏 can be expressed as:


(2) $$\tau \;=\;t_{1/2}\;-\;(V_{m}\;-\;V_{i})/(2\times k_{1/2})$$

A double-exponential model was selected as it provided a significantly improved fit over a single-exponential model (Supplementary Figure S1). For simplicity, equal weighting was applied to the two exponential terms, resulting in an excellent fit with minimal residuals.

The maximum aggregation rate (k_max_) and the time (*η*) at which k_max_ occurs were determined by calculating the first and second derivatives of the kinetic curves. Specifically, *η* represents the time at which the second derivative of the kinetic curve first reaches zero after the initial peak, marking the onset of the fastest aggregation rate. k_max_ is derived from the first derivative of the kinetic curve at time *η*. To minimize noise, a running average was applied to the kinetic data, with averaging intervals of 10 s for the aggregation data, 10 s for the first derivative, and 20 s for the second derivative. All derivative calculations were based on these smoothed data. Mean values and standard deviations for all kinetic parameters were obtained from three independent measurements.

## Supplementary material

online supplementary material 1

## Data Availability

Authors agree to make any materials, data, and associated protocols available upon request.

## Open Access

This article has been published open access under our Subscribe to Open programme, made possible through the support of our subscribing institutions, learn more here: https://portlandpress.com/pages/open_access_options_and_prices#conditional

## Abbreviations

Aβ: amyloid-β
CBD: calcium-binding C-terminal domain
Ca^2+^: calcium
EGTA: ethylene glycol tetraacetic acid
ER: endoplasmic reticulum
LED: light-emitting diode
MDR: multidrug resistance
NTD: N-terminal domain
PDB: Protein Data Bank
PMT: photomultiplier tube
RyRs: ryanodine receptors
SERCA: sarco/endoplasmic reticulum Ca^2+^ ATPase
TEV: Tobacco Etch Virus
ThT: thioflavin-T
Trap1: tumor necrosis factor receptor-associated protein 1
V_i_: initial scattering signal
V_m_: maximum scattering signal
k_max_: maximum aggregation rate
t_1/2_: half-time
βME: β-mercaptoethanol
τ: lag time
