# Supplementary material for: Kinetic investigation of calcium-induced Sorcin aggregation by stopped-flow light scattering
Source: Biochem J. 2025 Oct 28;482(21):1677–88. doi: 10.1042/BCJ20253194 (PMC12687447; doi:10.1042/BCJ20253194)
Supplement: online supplementary material 1 [file bcj-482-21-BCJ20253194-s001.docx]

**Supporting Information for**

**Kinetic investigation of calcium-induced Sorcin aggregation by stopped-flow light scattering**

Qiushi Ye^1,2,3^, Kathleen Joyce Carillo^2,3^, Nicolas Delaeter^2^, Lei Zhang^1^, Jaekyun Jeon^2^, Yanxin Liu^2,3,*^

^1^ School of Physics, Xi’an Jiaotong University, Xi’an, Shaanxi, 710049, China

^2^ Institute for Bioscience and Biotechnology Research, University of Maryland | National Institute of Standard and Technology, Rockville, MD 20850, USA

^3^ Department of Chemistry and Biochemistry, University of Maryland, College Park, MD 20740, USA

^*^Corresponding author: [yxliu@umd.edu](mailto:yxliu@umd.edu)


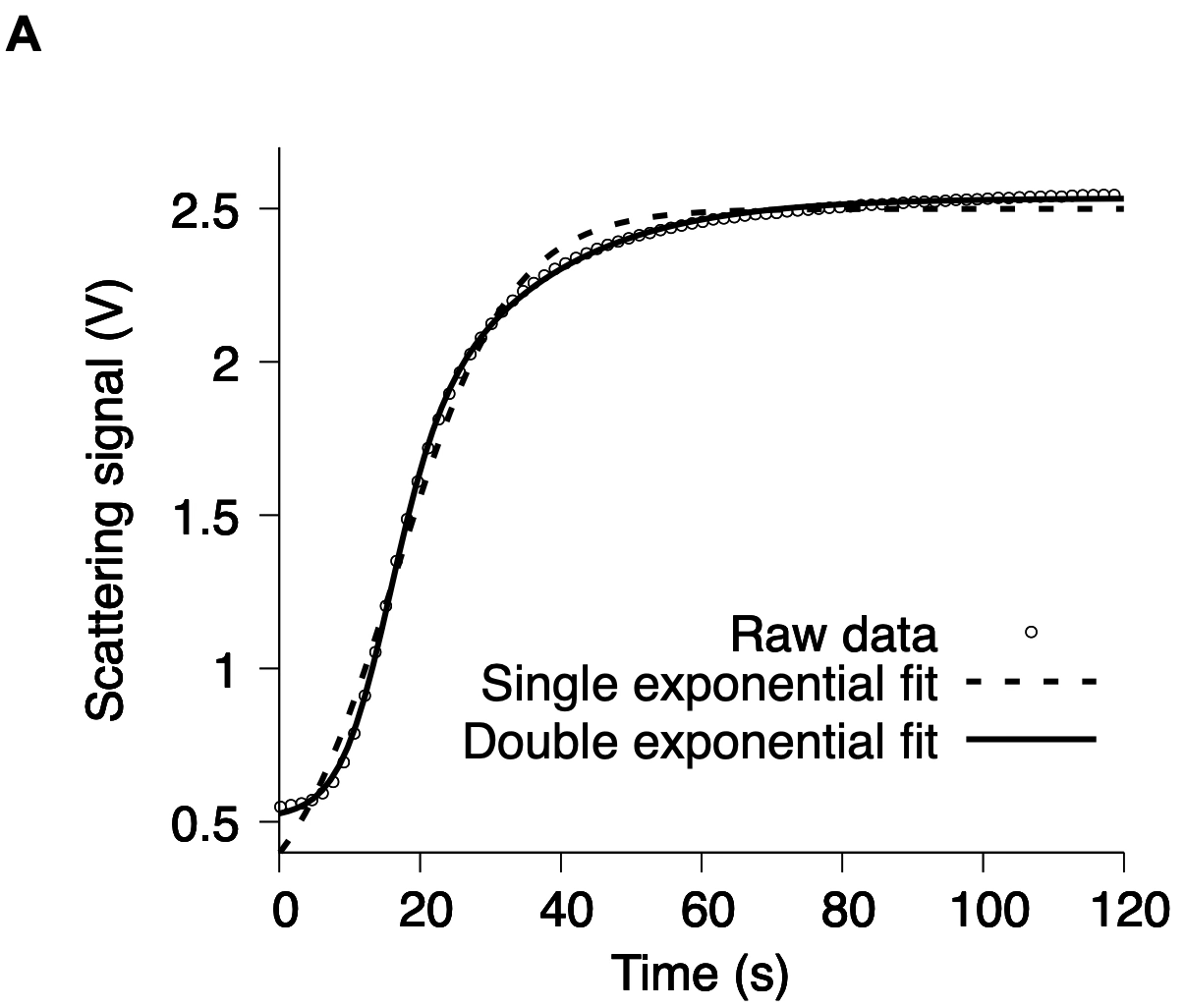


**Figure S1. Comparison between single and double exponential fits to Sorcin aggregation kinetics.**The light scattering kinetics trace (circles) was obtained by rapidly mixing 1 µM Sorcin with 150 µM Ca^2+^using stopped flow. The dashed line represents the single-exponential fit, yielding a root mean square deviation (RMSD) of 0.047 between the raw data and the fit. The solid line represents the double-exponential fit, yielding a significantly lower RMSD of 0.009, indicating a better fit to the data.


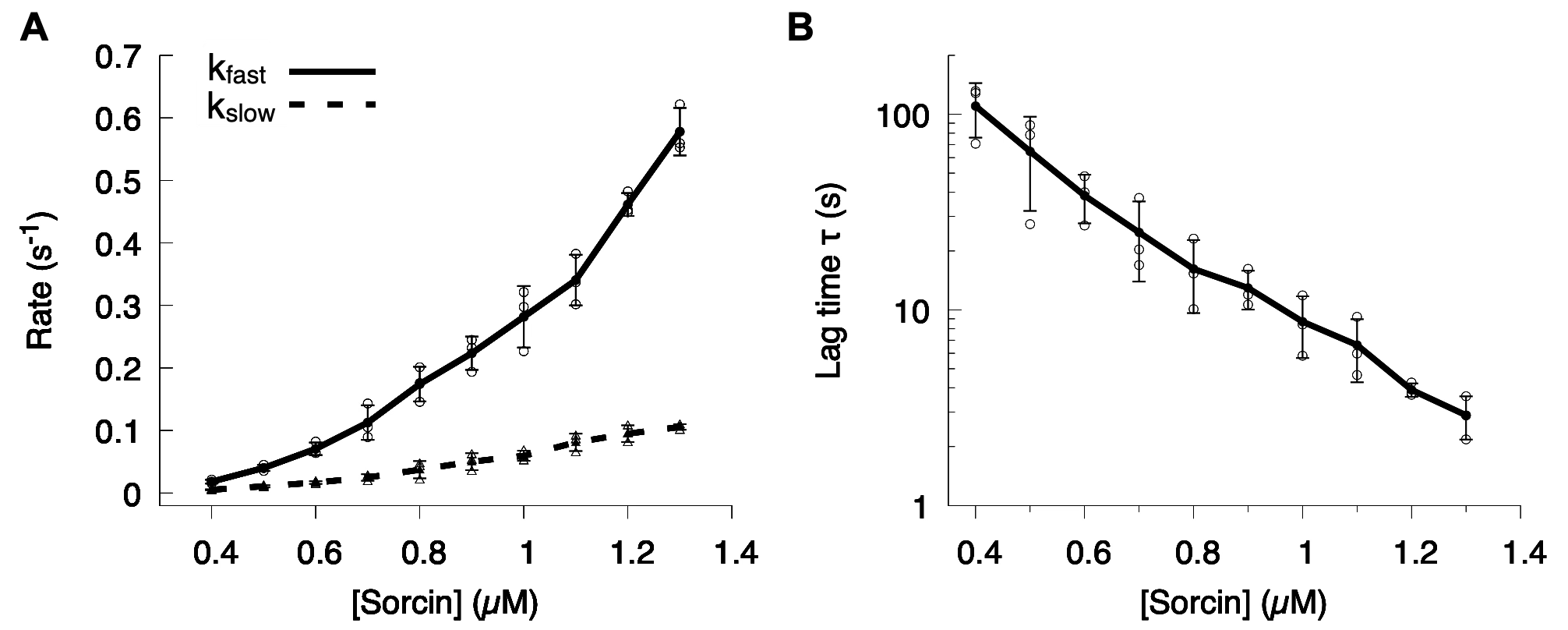


**Figure S2. Dependence of the aggregation growth rates (k_fast_ and k_slow_)** and **lag time τ on protein concentration.** (A) The aggregation growth rates (k_fast_​, and k_slow_) as a function of Sorcin concentration are shown as circles. The **k_fast_ and k_slow_** values at each concentration are connected by solid and dashed lines, respectively. (B) The τ values, calculated from three independent experiments at each Sorcin concentration, are shown as circles, and the average τ at each concentration is connected by a solid line.


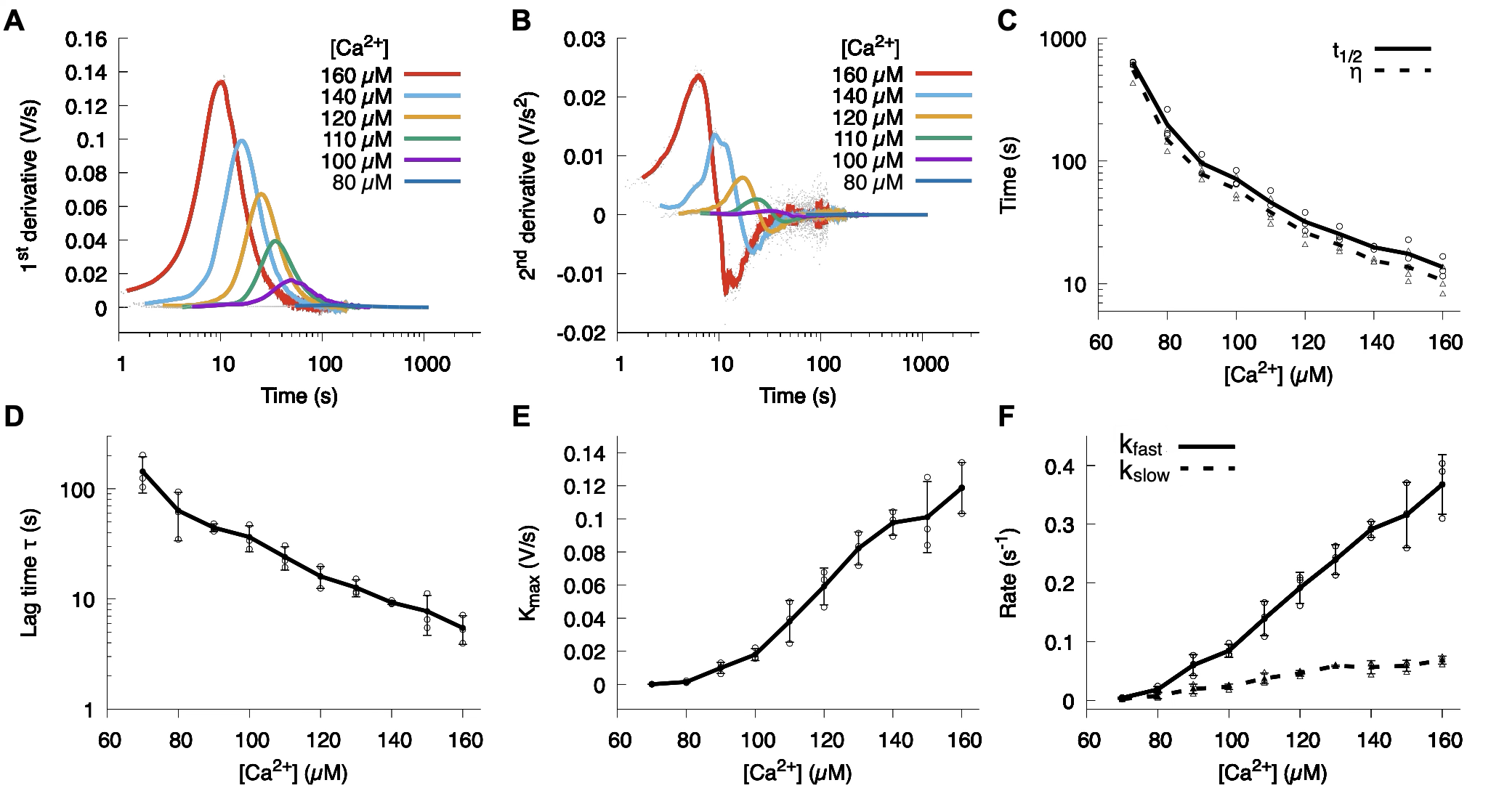


**Figure S3. Sorcin aggregation kinetics depend on Ca^2+^ concentration.** (A) First derivative of representative aggregation kinetics curves at varying Ca^2+^ concentrations. (B) Second derivative of these curves, with raw data shown as gray dots and a running average (solid line) applied to reduce noise. (C) Both the aggregation half-time (t_1/2_) and the time at which aggregation reaches its maximum rate (η) depend on Ca^2+^ concentration. (D) The lag time (τ) depends on Ca^2+^ concentration. (E) The maximum aggregation growth rate (k_max_) depends on Ca^2+^ concentration. (F) The aggregation growth rates (k_fast_​ and k_slow_) depend on Ca^2+^ concentration. Data were collected from three independent replicates at each Ca^2+^ concentration. The extracted kinetic parameters were plotted from each experiment as circles or triangles. The averages from these three replicates were connected by solid and dash lines to show the trend.


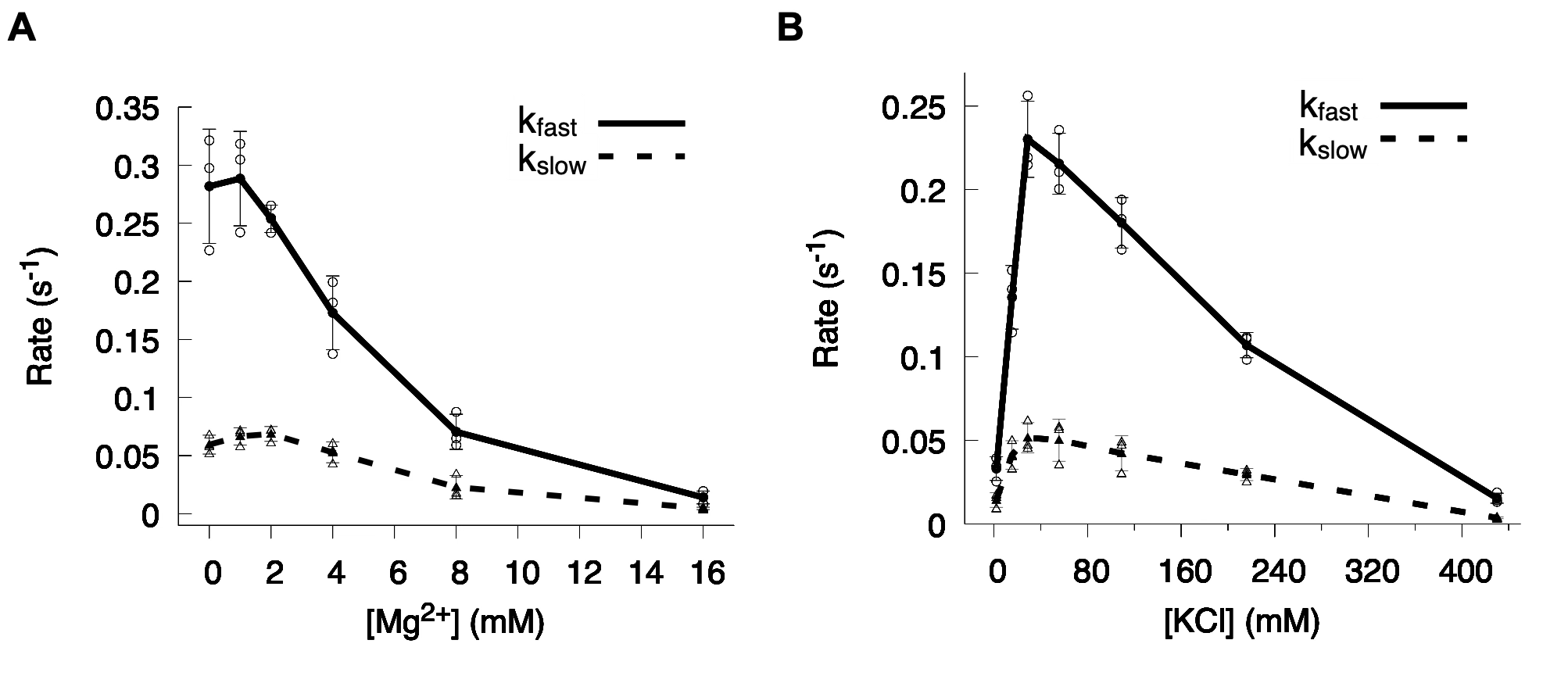


**Figure S4. Effect of Mg^2+^ and salt concentrations on the rate of Sorcin aggregation.** (A) The Sorcin aggregation growth rates (​k_fast_​ and k_slow​_) decrease as Mg^2+^ concentration increases. (B) Dependence of Sorcin aggregation growth rates (​k_fast_​ and k_slow​_) on KCl concentration. Data were collected from three independent replicates at each experimental condition. The extracted kinetic parameters (​k_fast_​ and k_slow​_) were plotted from each experiment as circles and triangles, respectively. The averages were calculated from these three replicates and connected by a solid and dash lines, respectively, to show the trend. The standard deviations were shown as the error bar.


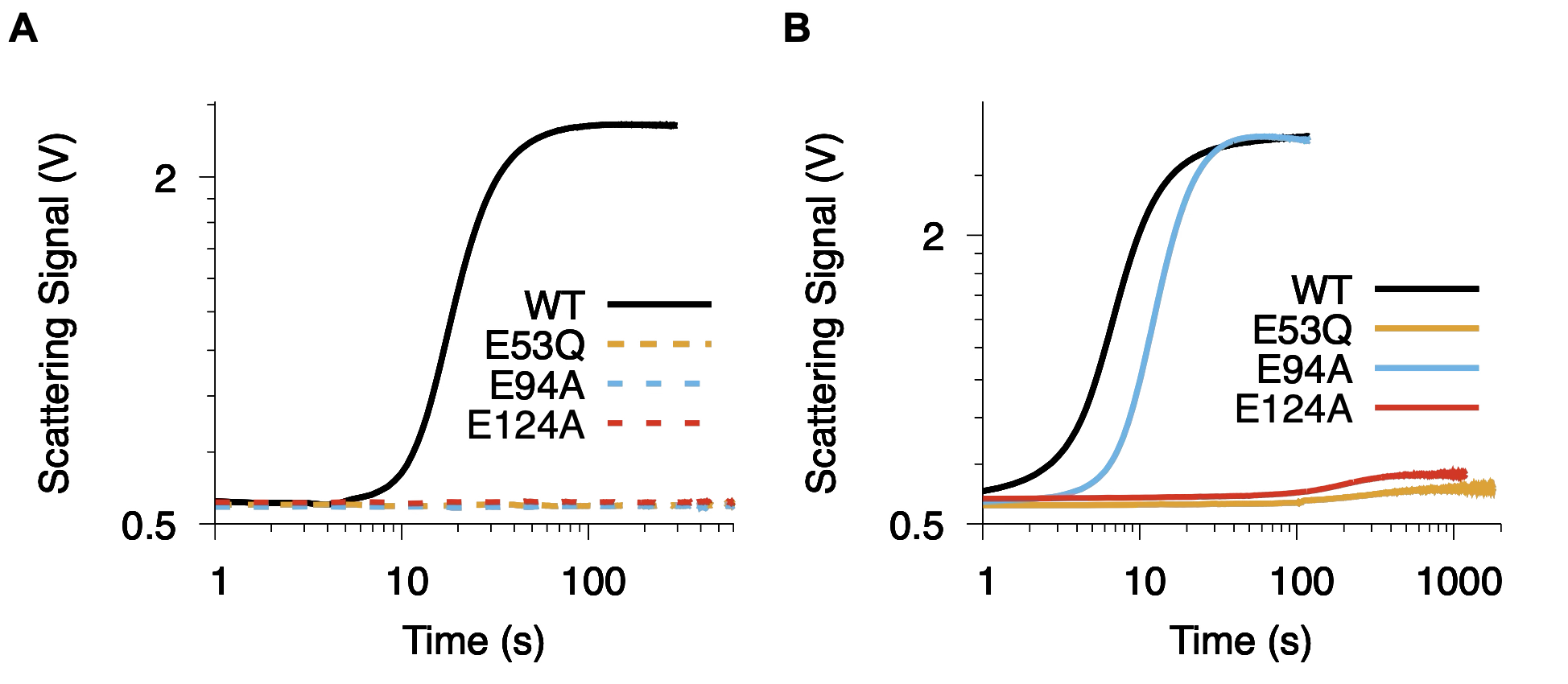


**Figure S5. Effect of mutations in three EF hands on Sorcin aggregation.** (A) Representative Sorcin aggregation kinetics curves for 1 µM Sorcin (wildtype or EF hand mutants) mixed with 150 µM Ca^2+^. No aggregation was observed for the EF hand mutants under these conditions. (B) Representative Sorcin aggregation kinetics curves at increased Sorcin and Ca^2+^ concentrations. The specific conditions are as follows: wildtype Sorcin (1.3 µM Sorcin, 150 µM Ca^2+^), E53Q and E94A mutants (1.3 µM Sorcin, 1 mM Ca^2+^) and E124A mutant (2 µM Sorcin, 2 mM Ca^2+^).
